# Nature of the Association between Rheumatoid Arthritis and Cervical Cancer and Its Potential Therapeutic Implications

**DOI:** 10.3390/nu16152569

**Published:** 2024-08-05

**Authors:** Kirill Gusakov, Alexander Kalinkovich, Shai Ashkenazi, Gregory Livshits

**Affiliations:** 1 Department of Morphological Sciences, Adelson School of Medicine, Ariel University, Ariel 4077625, Israel; kirilg@ariel.ac.il (K.G.); shaias@ariel.ac.il (S.A.); 2 Department of Anatomy and Anthropology, Faculty of Medical and Health Sciences, Tel-Aviv University, Tel-Aviv 6905126, Israel; kalinkovich@tauex.tau.ac.il

**Keywords:** HPV-infection, nutrition, chronic inflammation, dysbiosis, specialized pro-resolving mediators

## Abstract

It is now established that patients with rheumatoid arthritis (RA) have an increased risk of developing cervical cancer (CC) or its precursor, cervical intraepithelial neoplasia (CIN). However, the underlying mechanisms of this association have not been elucidated. RA is characterized by unresolved chronic inflammation. It is suggested that human papillomavirus (HPV) infection in RA patients exacerbates inflammation, increasing the risk of CC. The tumor microenvironment in RA patients with CC is also marked by chronic inflammation, which aggravates the manifestations of both conditions. Gut and vaginal dysbiosis are also considered potential mechanisms that contribute to the chronic inflammation and aggravation of RA and CC manifestations. Numerous clinical and pre-clinical studies have demonstrated the beneficial effects of various nutritional approaches to attenuate chronic inflammation, including polyunsaturated fatty acids and their derivatives, specialized pro-resolving mediators (SPMs), probiotics, prebiotics, and certain diets. We believe that successful resolution of chronic inflammation and correction of dysbiosis, in combination with current anti-RA and anti-CC therapies, is a promising therapeutic approach for RA and CC. This approach could also reduce the risk of CC development in HPV-infected RA patients.

## 1. Introduction

Patients suffering from autoimmune rheumatic diseases have an increased risk of developing cancer compared to the general population. In patients with rheumatoid arthritis (RA), the risk of developing solid tumors is approximately 20% higher than in the general population [1]. There are notable differences with respect to the type of solid cancer emerging in RA patients. There is increased incidence of solid tumors associated with smoking or oncogenic viral infections, such as lung, bladder, kidney, upper urinary tract, and cervical cancer (CC), and decreased incidence of breast and colon cancer [2]. Studies published in the past decade have revealed a 1.5–3.65 times higher risk of developing CC in RA patients infected with human papillomavirus (HPV) [3,4,5]. In this regard, the main question is the following: what mechanisms are responsible for this pathological phenomenon? By answering this question, we can develop therapeutic measures to prevent the development of CC in HPV-infected RA patients. 

RA is a multifactorial autoimmune disease in which the main pathogenic mechanism is unresolved chronic inflammation [2]. It presumably creates conditions favorable to the development of CC in HPV-infected RA patients. Another frequently suggested risk factor is the use of immunosuppressive drugs to treat RA, such as disease-modifying antirheumatic drugs (DMARDs). However, the majority of DMARDs are not associated with increased cancer risk in patients with systemic rheumatic diseases, including RA [1]. Noticeably, chronic inflammation is a common characteristic of various solid cancers [6]. HPV infection has been repeatedly shown to induce and maintain chronic inflammation, predominantly observed in the CC tumor microenvironment (TME) [7]. The molecular components of chronic inflammation associated with RA and CC share similarities. These include activated “classical” signaling pathways, such as nuclear factor kappa B (NF-kB), Janus kinase/signal transducers and activators of transcription (JAK-STAT), mitogen-activated protein kinase (MAPK), and toll-like receptor (TLR) pathways [8,9]. All these pathways cause the production of a variety of pro-inflammatory, tissue-damaging factors, such as cytokines, chemokines, and metalloproteinases (MMPs) [7]. In both RA and CC, activated inflammasomes [10,11] and the cyclic GMP-AMP interferon synthase stimulator gene (cGAS–STING) signaling pathway [12,13] are involved in the development of chronic inflammation.

Dysbiosis, an imbalanced microbial community, is considered a strong factor in inducing inflammation in a wide variety of diseases, including RA and CC [14,15]. Moreover, recent Mendelian randomization studies have suggested causal links between certain components of gut microbiota and RA [16,17] and CC risk [18,19]. These data suggest using dysbiosis-correcting therapeutic approaches in an attempt to mitigate RA and CC manifestations. In support, in pre-clinical and clinical studies, probiotics, Mediterranean diet, and other nutritional regimens, including polyunsaturated fatty acids (PUFAs) and their derivatives, specialized pro-resolving mediators (SPMs), have demonstrated beneficial effects on the manifestations of RA [20] and CC [21]. For example, vitamin D supplementation and dietary sodium restriction have been shown to be beneficial for some RA outcomes. In addition, fasting resulted in significant but temporary subjective improvements [20]. The most important role of diet and nutrition in relation to CC is the prevention and suppression of HPV infection. The main preventive and restorative factors for CC are antioxidants, such as vitamins A, C, D, and E, and carotenoids, found in vegetables and fruits [22]. Resolution of chronic inflammation is suggested as a key mechanism underlying the favorable effects of these dietary or supplementary regimens [23,24].

CC ranks as one of the most common malignancies in females [25]. Persistent HPV infection is the primary risk factor for CC. While most HPV-infected women clear the virus within several months, only a small percentage of those infected with HPV eventually develop precancerous cervical intraepithelial neoplasia (CIN) or CC [26]. However, the exact molecular mechanisms of HPV clearance or the progress to cancer from the pre-cancer stage remain poorly understood [27,28,29]. A recent spatial transcriptomics study found that several upregulated genes are associated with the transition from pre-cancer to CC in HPV-infected cells. These genes include KRT6C, KRTDAP, and S100A7, which are closely linked to the processes of carcinogenesis, invasion, and migration [30]. 

Based on all these observations, we hypothesize that unresolved chronic inflammation and certain genetic factors maintain and promote RA and CC manifestations, and also underlie the increased risk of CC in HPV-infected RA patients. We believe that the effective resolution of chronic inflammation could attenuate the manifestations of both diseases and decrease CC development in HPV-infected RA patients. Evidence supporting this concept prompted the present review.

## 2. HPV-Induced CC Pathogenesis

Although CC etiology and pathogenesis are the subject of extensive research, several key aspects of its pathogenesis, in particular the role of genetic and inflammatory factors, remain poorly understood. It is well established that persistent HPV infection is the primary risk factor for developing CC. However, only a small percentage of those infected with HPV eventually develop precancerous lesions or cancer [26]. 

HPV is a double-stranded cyclic DNA virus. It has early (E), late (L), and upstream regulatory (URR) gene regions. The E region consists of E1 to E7 genes responsible for viral replication. The E1 and E2 proteins play a role in virus replication and translation. E2 also regulates E6 and E7 expression, and the E4 and E5 proteins mediate viral assembly and stimulate growth [26]. The main carcinogenic mechanism is attributed to the ability of oncoproteins E6 and E7 to bind to and break down tumor suppressor proteins, promoting viral replication and inhibiting host cell apoptosis [31]. Upon binding to E6-associated protein, E6 inhibits the tumor-suppressor p53 gene. The LxCxE sequence of E7 binds to the Retinoblastoma protein (Rb), which then undergoes phosphorylation, and its function is inhibited. This leads to uncontrolled regulation of the cell cycle G1 phase. E6 and E7 activate the CTGF, CYR61, and FOXM1 genes, promoting tumor cell growth, blood vessel formation, and immune system evasion. Regardless of HPV infection, CC carcinogenesis is characterized by loss of function mutation in the TP53 gene, which encodes the tumor-suppressor p53 protein, resulting in genetic instability, cellular immortalization, accumulation of mutations, and the eventual malignant transformation of cells [31]. Our proteome profiling of cervicovaginal fluid of HPV-infected women has revealed 27 significantly altered proteins in CC patients compared with precancerous patients [32]. Some of these identified genes, such as ACTN4, VTN, ANXA1, CAP1, ANXA2, and MUC5B, have been proposed as CC biomarkers [33]. CIN, for which HPV infection is a causal factor, represents a non-malignant precancerous stage that has the potential to progress to CC. This transition is also accompanied by significant changes in gene expression levels, including PIK3CA, FBXW7, MAPK1, NRF2, TP53, ERBB2, PTEN, and KRAS [34].

The structure of the HPV genome is not very complex. The product of each viral gene is a highly specialized protein that mediates processes of viral attachment to specific cellular receptors, cellular entry, alternative splicing, gene interaction, and expression of both viral and host cell proteins. Combined, these viral processes promote uncontrolled cell division by regulating various signaling pathways and bypassing host defense mechanisms, including downregulation of TLR9 expression in infected cells [35], suppression of nuclear factor-kappa B (NFκB) activity [36], or inhibition of type I interferon (IFN) pathways [37].

## 3. Epidemiological Association between RA and CC

Recent epidemiological studies consistently indicate an increased prevalence of CC and CIN in patients with RA [1,3,4,38,39]. However, a few previous studies have not supported this conclusion (e.g., [40]). Table 1 summarizes these epidemiological studies; despite differences in sample size, ethnicities, cultures, and demographics, a consistent trend suggests a positive association between RA and CC.

An Australian retrospective cohort study shows an increased risk of low-grade CIN, with an estimated adjusted hazard ratio (HR) of 1.23 (95% CI 1.05–1.42) [39]. Similar estimates have been obtained in a nationwide registry-based cohort study in Sweden [4]. It estimates a significant risk of CIN 2–3 (HR 1.39; 95% CI 1.16–1.66) in women with RA, who do not receive biologic DMARDs (treated with at least two of the following drugs: methotrexate, sulfasalazine, antimalarials, or leflunomide), and a significant risk of CC (HR 2.10, 95% CI 1.04–4.23) in the tumor necrosis factor inhibitor (TNFi) treatment group. In agreement, a Danish nationwide population-based registry study displays similar results, although the standardized incidence ratio (SIR) for CC did not achieve a statistically significant value: 1.1 (95% CI 0.9–1.2) [38]. In contrast, a very large French nationwide population-based study of 189,335 women with RA reports a clearly elevated prevalence of CC (SIR 1.80; 95% CI 1.62–2.01) [1]. Importantly, the risk of CC is higher independently of the type of RA treatment: the greatest observed risk (SIR 2.28; 95% CI 1.22–4.26) is in anti-interleukin-6 receptor antibody therapy group, while in the Abatacept and Rituximab groups it is the smallest (SIR 1.32; 95% CI 0.54–3.19 and 1.35; 95% CI 0.56–3.26, respectively). In accordance, a large cohort study of US women with systemic inflammatory diseases reports an elevated prevalence of CC and high-grade CIN in RA patients (HR 1.49; 95% CI 1.11–2.01) [3]. The researchers attribute these findings to differences in the severity of systemic inflammation or the use of systemic immunosuppressive drugs or steroids. Notably, the RA–CC association is not limited to the Caucasian population and has also been observed in the Asian population. A Korean retrospective cohort study of 1586 women with RA demonstrates a marked increase in CC risk (SIR 3.65; 95% CI 1.65–6.42) [5]. There is no difference in the use of DMARDs between cancer and non-cancer groups among the RA patients, with the exception of hydroxychloroquine (78.4% of use in the non-cancer group against 68.0% in the cancer group). 

One may assume that increased CC incidence could be a result of RA patients being more vigilant about participating in cancer screening programs following an RA diagnosis. According to this notion, an increased breast cancer incidence would have also been detected among RA patients, as a breast cancer screening program was also available. However, these studies provide no evidence of increased breast cancer prevalence in RA patients, despite years of the screening program [1]. Paired with the fact that all the countries, where the aforementioned epidemiological studies were conducted, have implemented widespread HPV vaccination, the trend for the association between RA and CC becomes especially concerning.

While screening and prevention programs for HPV-associated CC have proven to be effective, the data provided in the present review indicate that RA patients have an increased risk of CC development. Currently, there are no guidelines that differentiate RA patients as a group that requires an individualized screening program [41,42,43]. RA patients should be encouraged to receive the HPV vaccine according to national vaccination programs to reduce the risk of CC [44]. Educating patients about the importance of vaccination is crucial, especially since they may be at higher risk for HPV-related diseases. Medical experts should actively educate RA patients about CC screening programs and encourage their participation. Regular screenings are vital for early detection and improved outcomes [45,46]. In addition, while currently there are no specific dietary supplements suggestions in the guidelines, dietary alterations may be beneficial for RA patients. Possible recommendations may include certain diets (e.g., Mediterranean diet), calorie restriction, and the supplementation of PUFAs, polyphenols, and vitamins (see below).

In conclusion, despite some controversial results published earlier, the recent large studies convincingly show a significantly elevated prevalence of both CC and CIN 2+ in RA patients (Table 1). The critical question is, what is the nature of this association? Since both diseases are multifactorial, caused by a variety of extrinsic and intrinsic factors, we will herewith discuss the major ones. We will first focus on the impact of the gut and vaginal microbiomes, as there is little doubt that dysbiosis plays a significant role in the etiology of very diverse illnesses, including RA and different types of cancers. Furthermore, there is growing evidence that diet has a profound effect on shaping the microbiota, which can introduce beneficial effects on the progression of these diseases. We will also discuss how nutrition affects the activation of the inflammasome and the cGAS–STING signaling pathway in the pathogenesis of RA and CC.

## 4. Nutrition and the Role of Gut Dysbiosis in RA Manifestations

Gut dysbiosis is a well-known key factor that triggers and maintains chronic inflammation in RA [47,48,49,50,51,52]. Remarkably, it occurs several years before the radiographic confirmation of RA and its autoimmune and inflammation manifestations [53]. This suggests an important role for the microbiota in the transition from the preclinical to clinical stage of RA [54]. The current evidence indicates that RA patients can be distinguished from healthy individuals based on alterations in the composition of their gut microbiota, similar to animal RA models compared to their healthy counterparts [14,55]. A microbiome unique to RA patients has not been identified. However, certain bacteria, mainly *Prevotella*, *Citrobacter rodentium*, *Collinsella aerofaciens*, and *Segmented filamentous*, have been found to contribute to the creation of the pro-inflammatory immune status, thus playing a role in the development and maintenance of RA [55,56]. These effects presumably involve bacterial mechanisms of epitope mimicry, modification of self-antigens, enhanced cell apoptosis, and destruction of tight junction proteins and intestinal barrier integrity. However, not all species of microbiota in the *Prevotella* genus are pro-inflammatory. For example, *Prevotella histicola* has been shown to reduce arthritis severity in mice with collagen-induced arthritis [57], and several other studies have shown that *Prevotella* species are among the most common commensal bacteria in healthy humans and exhibit beneficial properties for the host [58].

Interestingly, two recent Mendelian randomization studies suggest a causal relationship between gut dysbiosis and the risk of RA [17,59]. Certain bacteria species have revealed wither protective effects (e.g., *Alloprevotella*, *Christensenellaceae*, *Paraprevotella*, and *Gordonibacter* groups) or enhancing effects (e.g., *Ruminococcaceae* and *Parabacteroides* groups).

**Figure 1:**
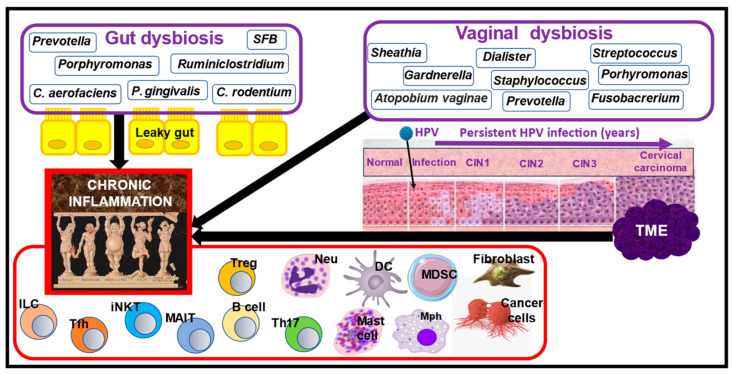
Schematic presentation of mechanisms underlying the involvement of gut and vaginal dysbiosis in the creation of chronic inflammation in RA patients with HPV-induced CC. Gut and vaginal dysbiosis in RA patients is characterized by the prevalence of specific bacteria species that cause several detrimental effects, such as the destruction of tight junction proteins and intestinal barrier integrity. This results in lipopolysaccharide leakage into the gut lumen, leading to the activation of gut-resident immune cells, including dendritic cells (DCs), macrophages, neutrophils, intraepithelial lymphoid cells (ILCs), Th17 cells, B cells, invariant natural killer T cells (iNKTs), mucosal-associated invariant T cells (MAITs), T-follicular helper cells (Tfhs), and T-regulatory cells (Tregs). The activated immune cells secrete a variety of pro-inflammatory molecules, which can activate epithelial and immune cells in autocrine, paracrine, and endocrine (systematic) manners, culminating in the creation of chronic inflammation. HPV infection is accompanied by both gut and vaginal dysbiosis, typically associated with the prevalence of bacterial species, as displayed in the diagram. Development of CC in HPV-infected RA patients exacerbates gut and vaginal dysbiosis. Dysbiosis, in turn, maintains and worsens chronic inflammation, which, as suggested in the review, is responsible for the aggravation of RA and CC manifestations. Further explanations are given in the text. *Abbreviations*: CIN, cervical intraepithelial neoplasia; *C. rodentium*, *Citrobacter rodentium*; *C. aerofaciens*, *Collinsella aerofaciens*; HPV, human papillomavirus; *P. gingivalis*, *Porphyromonas gingivalis*; SFB, segmented filamentous bacteria; TME, tumor microenvironment.

Pathogenic bacteria (pathobionts) and their products, mainly lipopolysaccharide (LPS), activate the gut-associated lymphoid tissue (GALT) located in the intestinal wall [60]. The main cellular components of the GALT are innate lymphoid cells, mucosa-associated invariant T cells, invariant natural killer T cells, T-follicular helper cells, and regulatory T cells [61]. Other immune cells in the lamina propria include interleukin-17-secreting T helper cells (Th17s), B cells, dendritic cells (DCs), macrophages, and neutrophils. When these cells become active, they secrete a variety of pro-inflammatory cytokines and autoantibodies capable of activating epithelial and immune cells in autocrine, paracrine, and/or endocrine manners. This inflammatory process generates a deleterious hierarchical cytokine network that ultimately drives the development of RA-associated joint inflammation and bone destruction [49]. Moreover, gut-related immune dysfunction has been shown to promote the overgrowth of bacteria, which maintain RA through a harmful vicious cycle [14,49,54,58,62,63,64]. These events are schematically presented in Figure 1. 

The involvement of gut dysbiosis in RA pathogenesis suggests that restoring normal microbiota can potentially reduce the transition from the asymptomatic phase to the clinical symptomatic phase. Accumulating evidence implies that appropriate nutrition could facilitate such beneficial effects. Common dietary approaches to positively impact the gut microbiota include the Mediterranean diet, vegetarian/vegan diets, gluten-free diet, vitamin D supplementation, elemental/elimination diets, intermittent fasting, and the use of dietary supplements, especially PUFAs or fish oils [65]. However, in a series of meta-analyses, only the Mediterranean diet has demonstrated improvements in some measures of RA disease symptoms, whereas other dietary approaches have been controversial [20,66,67]. Taking omega-3 PUFAs in high doses leads to a decrease in RA disease symptoms and pharmacotherapy failure rates [68,69,70].

Among other food supplements used to alleviate RA symptoms are probiotics, defined as “live microorganisms that, when administered in adequate amounts, confer a health benefit on the host” [71]. The most commonly used probiotics contain lactic acid-producing bacteria, such as *Lactobacillus* and *Bifidobacterium* [72]. The mechanisms of action of probiotics include mucus secretion, production of vitamins and short-chain fatty acids (SCFAs), antimicrobial peptides, maintenance of the epithelial barrier function, and reduction in oxidative stress. They are involved in the response of the host immune system to pathobionts, mainly via the interaction of SCFAs with DCs [14,49,73,74]. The use of probiotics has demonstrated positive effects in pre-clinical arthritis models [75,76]. Several clinical studies have also indicated beneficial effects in RA patients (reviewed in [76]). However, recent meta-analyses of clinical trials using probiotics for RA treatment found that most studies involved small cohorts and were short-term, leading to high variability in the lab and clinical results, with inconsistent therapeutic outcomes [77,78,79,80].

There is growing published data that consuming dietary fiber intake decreases the risk of various diseases, including coronary artery disease, and pancreatic and gastric cancers, presumably through modulation of the gut microbiota [81]. A recent study shows that consuming fiber intake is negatively associated with high-sensitivity C-reactive protein levels and a reduced RA risk, confirming the benefit of fiber intake in slowing the progression of RA [82]. Collectively, these observations indicate gut dysbiosis plays a significant role in the pathogenesis of RA, the symptoms of which can be alleviated by the use of various dietary approaches.

## 5. Nutrition and the Role of Vaginal and Gut Dysbiosis in CC Pathogenesis

There is a substantial body of studies that point to the relationship between vaginal [83] and gut [15,83] dysbiosis and the risk of CC. Healthy women typically have over 200 bacterial species in their vaginal flora, mainly dominated by one of the four most prevalent *Lactobacillus* species (*Lactobacillus crispatus* (*L. crispatus*), *LL. iners*, *L. gasseri*, and *L. jensenii*) [84,85]. Persistent HPV infection and cervical carcinogenesis are accompanied by a pronounced vaginal dysbiosis, characterized by a decrease in the abundance of *Lactobacillus* species and enrichment of *Sneathia, Gardenella, Prevotella*, and other bacteria [83,86]. These vaginal flora alterations are considered one of the main factors in causing and maintaining chronic inflammation associated with HPV [87,88,89]. Compared to healthy individuals, CC patients with vaginal dysbiosis exhibit increased vaginal mRNA levels of the pro-inflammatory cytokines interleukin-1α (IL-1α), IL-1β, IL-6, IL-8, and tumor necrosis factor α (TNFα) and decreased vaginal levels of the anti-inflammatory cytokines IL-4 and transforming growth factor-β1 (TGF-β1) [90]. In CC, chronic inflammation is predominantly present in TME, where various immune cells, cancer cells, myeloid-derived suppressor cells (MDSCs), mast cells, and fibroblasts secrete a variety of immunosuppressive and pro-inflammatory molecules, thereby perpetuating HPV-induced chronic inflammation (Figure 1). 

The aforementioned significant differences in vaginal microbiota between HPV-free/cleared women and women with persistent infection raise the question of a cause-and-effect relationship. This relationship, like the one between dysbiosis and RA pathogenesis, is a subject for research. It has been recently proposed that specific microbiota may influence the course of a previously acquired HPV infection rather than the likelihood of contracting a new HPV infection [91]. Thus, the consequences of HPV infection can be determined by the cervical microbiota. Of interest, the prophylactic effect of response to the HPV vaccine may be related to the composition of the vaginal microbiota [92].

A recent study demonstrates significant differences in the gut microbiota composition between healthy controls and CC patients, highlighting the relationship between gut dysbiosis and CC [15]. In particular, the disease stage and prognosis were most significantly negatively correlated with *Ruminococcus* 2, a bacterium associated with various types of inflammation. A recent bidirectional Mendelian randomization study has proposed a causal relationship between gut dysbiosis and CC development, in which certain bacteria classes predict a higher or lower risk of CC [18]. These observations suggest that correcting vaginal and gut dysbiosis can slow down CC progression. Various diets, probiotics, and prebiotics have been given to patients with vaginal and gut dysbiosis. The use of antioxidants, such as vitamin A, C, D, and E, carotenoids, vegetables, fruits, and Mediterranean diet are recommended for preventing and clearing HPV infection [22]. Additionally, vaginal application of probiotics like *Lactobacillus casei* (*L. casei*), *L. rhamnosus*, *L. reuteri*, *L. acidophilus*, *L. plantarum*, *L. crispatus*, and *L. gasseri*, along with oral intake of probiotic *L. crispatus*, is recommended [93]. To avoid intestinal and vaginal dysbiosis during CC radiation/chemotherapy, it is recommended to take *L. acidophilus* and *Bifidobacterium bifidum* orally, along with dietary fiber and Mediterranean diet [93]. A consensus statement from the European Society of Gynecological Oncology argues that although prebiotics and probiotics have been reported to be beneficial in gynecological malignancies, including CC, studies are small and of variable design; therefore, high-quality evidence to support their use is still lacking [94].

Another aspect in the role of dysbiosis in the treatment of CC and RA is the potential clinical efficacy of fecal microbiota transplantation (FMT). In human cancer, this method has not yet been used as an independent therapeutic approach, but only in combination with immunotherapy represented by immune checkpoint inhibitors (ICIs) (reviewed in [95,96,97]). In addition to FMT, it has also been shown that vaginal microbiota transplantation (VMT) is a safe and effective approach in the treatment of bacterial vaginosis and other vaginal conditions, and to reduce the risk of sexually transmitted infections and preterm birth in pregnant women [98]. As for RA, there is only one case-report study of FMT from a healthy donor to refractory RA patient, which showed a good therapeutic effect with decreased arthritis index and rheumatoid factor level [99]. Two clinical trials evaluating the efficacy and safety of FMT for refractory RA are currently ongoing (NCT03944096 and NCT04924270), but the results have not yet been published (PMID: 36931952). Results of another recent clinical trial showed that FMT from healthy donors to patients with active systemic lupus erythematosus (SLE) is a safe and potentially effective procedure that alters the gut microbial community, modifies the SCFA metabolic profile in SLE patients, and reduces systemic immune inflammation profiles in SLE patients [53]. Collectively, these findings suggest translational potential of FMT/VMT in the treatment of severe cases of CC and RA patients with CC.

## 6. The Role of Chronic Inflammation in RA and CC Pathogenesis

**Figure 2:**
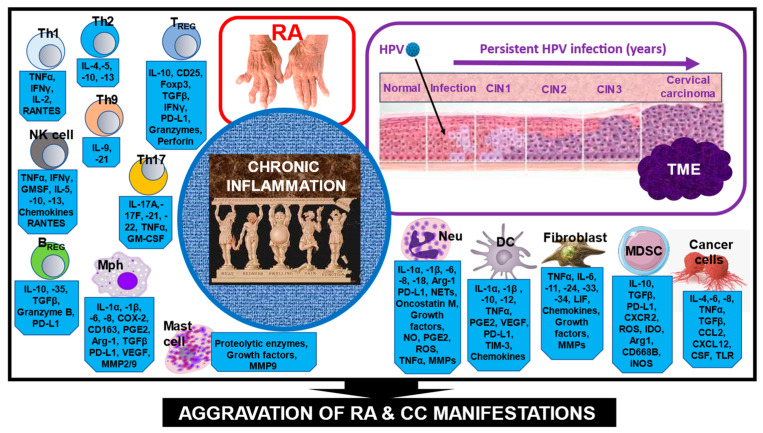
A schematic presentation of the chronic inflammatory profile in RA patients with CC. The cells depicted in the diagram are found in both the inflamed joints and the TME of HPV-infected RA patients with CC. They express and secrete a variety of factors, which are mostly pro-inflammatory or immunosuppressive, creating a vicious loop of chronic inflammation. Moreover, infection of RA patients with HPV can further aggravate chronic inflammation and manifestations. It can also contribute to the enhanced progression of CIN to CC. Further explanations are given in the text. *Abbreviations*: Arg-1, arginase 1; BREG, B regulatory cells; CCL20, chemokine ligand 20; COX, cyclooxygenase; CSF, colony-stimulating factor; CTLA, cytotoxic T lymphocyte-associated protein; DC, dendritic cell; Foxp3, forkhead box P3; GM-CSF, granulocyte-macrophage growth factor; HPV, human papillomavirus; IDO, indoleamine 2,3-dioxygenase; IFN, interferon; IL, interleukin; iNOS, inducible nitric oxide synthase; LIF, leukemia inhibitory factor; MDSC, myeloid-derived suppressor cells; MMP, metalloproteinase; Mph, macrophage; Nets, netosis; Neu, neutrophil; NK, natural killer cell; NO, nitrogen oxide; PD-L1, programmed death-ligand-1; PGE2, prostaglandin E2; RANKL, receptor activator of NF-kB ligand; RANTES, regulated upon activation, normal T cell expressed and presumably secreted; ROS, reactive oxygen species; TGF, transforming growth factor; Th, T helper cell; TLR, toll-like receptor; TME, tumor microenvironment; TREG, T regulatory cell; VEGF, vascular endothelial growth factor.

Dysbiosis in the gastrointestinal tract and vagina, which can be caused by various genetic and environmental factors, including HPV infection, exhibits bidirectional interactions with the host immune system. This immune response can have different consequences, particularly the development of an inflammatory disease [88,100] (Figure 2). Chronic synovial and systemic inflammation are the key pathogenic factors driving RA progression [101,102,103]. Activation of NF-kB, JAK-STAT, and MAPK pathways [104], as well as the activation of inflammasomes [105] and cGAS–STING pathway [106], are considered the main underlying inflammatory mechanisms in RA. In this inflammatory process, T cell subpopulations, such as Th1, Th9, and Th17 cells, produce pro-inflammatory cytokines, whereas other immune cells, such as Th2, Treg, and Breg cells, which normally secrete anti-inflammatory cytokines, are suppressed. DCs, monocytes/macrophages, and neutrophils also produce pro-inflammatory cytokines, chemokines, and other inflammatory factors. This causes resident cells in the joints, such as fibroblasts, chondrocytes, osteoclasts, and osteoblasts, to become activated and secrete pro-inflammatory soluble factors, ultimately recruiting immune cells into the joints. Altogether, these events create a strong and self-sustaining pro-inflammatory microenvironment [107,108]. Figure 2 schematically illustrates these events.

Chronic inflammation is a typical characteristic of HPV infection [109]. The viral oncoproteins E5, E6, and E7 promote chronic inflammation by increasing cyclooxygenase-2 (COX-2) expression and, consequently, prostaglandin E2 (PGE2) production [110]. Additionally, HPV can induce other inflammatory mediators, such as reactive oxygen and nitrogen species (ROS, RNS), as well as expression of the PGE2 receptor (PTGER) [111]. In turn, PTGER activation increases the release of inflammatory cytokines and MMPs, as well as COX-2 activity and expression [112]. In the CC/CIN-associated TME, N1-type neutrophils, M1-type macrophages, NK cells, DCs, and CD4+ and CD8+ T lymphocytes secrete cytokines, such as IL-2, IL-12, IL-18, and interferon-γ (IFNγ), as part of their combat against pre-malignant and malignant cells. In the advanced stages of CC development, N2-type neutrophils, M2-type macrophages, Th2 and Th17 lymphocytes, Treg and Breg cells, mast cells, MDSCs, as well as cancer cells, secrete various cytokines, such as IL-4, IL-10, IL-17, IL-35, and TGF-β. These cytokines suppress the activity of anti-cancer immune effector cells, thereby creating a microenvironment that is tolerant toward tumor cells, facilitating further tumor development [113] (Figure 2). Moreover, ongoing chronic inflammation during HPV infection is supported by high circulating levels of pro-inflammatory cytokines, such as IL-6, IL-1β, IL-8, TNFα, and MIP-1α [114]. 

Taken together, these findings convincingly show a significant role of chronic inflammation in the pathogenesis of both RA and CC. Notably, HPV infection can aggravate the manifestations of RA via a vicious loop of chronic inflammation. As postulated, this chronic systemic and vaginal inflammation is believed to potentially contribute to an accelerated progression from CIN to CC.

## 7. Nutrition and the Role of Inflammasome Activation in RA and CC Pathogenesis

As discussed above, the main component driving the pathogenesis of RA and CC is inflammatory immune activation. The inflammasome is an integral constituent of both the activated innate and adaptive immune systems. It serves a central intracellular sensor protein complex that detects a wide range of microbial motifs, endogenous danger signals, and various environmental stimuli [115,116]. Its activity is associated with a range of inflammatory disorders, including RA [117] and cancer [118]. Notably, nutritional components, such as polyphenols, fatty acids, proteins, carotenoids, vitamins, curcumin, resveratrol, and probiotics, have been shown to modulate inflammation by suppressing inflammasome activation [119]. Nucleotide-binding domain leucine-rich repeat (LRR)-containing (NLR) family members (NLRP1 and 3) are established components of the inflammasome [120]. Upon functional activation, the NLRP3 inflammasome triggers a caspase-1-dependent release of the pro-inflammatory cytokines IL-1β and IL-18, as well as gasdermin D (GSDMD)-mediated pyroptosis. The latter is a lytic form of inflammation-associated cell death, characterized by pore formation in the cell membrane that enables the extracellular release of IL-1β and IL-18 [120]. In addition, the NLRP3 inflammasome activates the NF-κB signaling pathway, which also plays a role in the production of IL-1β and IL-18 [121].

There is increasing evidence that the NLRP3 inflammasome plays a critical role in the pathogenesis of RA [11]. Indeed, the NLRP3 inflammasome is highly activated in the synovia and chondrocytes of RA patients, and pyroptosis is implicated in the onset and progression of RA [105]. The NLRP3 inflammasome is activated in CD4+ T cells of RA patients. This activation is associated with increased differentiation of Th17 cells, increased IL-17A production, and disease manifestations [122]. The NLRP3 inflammasome activates downstream TGF-β-activated kinase 1 (TAK1) in macrophages and osteoclasts, leading to the activation of synovial fibroblasts (SFs), which secrete IL-1β, thus orchestrating the inflammatory response [123]. The NLRP3 inflammasome mediates MMP production in SFs in response to TNFα and IL-6 and receptor activator of nuclear factor kappa-Β ligand (RANKL) production in response to IL-1β. Consequently, these factors promote the infiltration of inflammatory cells into the synovium as well as the destruction of cartilage and bone [123]. Of note, gene polymorphisms in the NLRP3 inflammasome have been observed to impact the susceptibility to RA and its severity; they can also determine therapeutic response rates to TNFα inhibitors in RA patients [124].

Given the pathogenic role of the highly activated NLRP3 inflammasome in RA, it has been considered a promising target for treating RA [125]. However, the as-yet-unknown clinical efficacy of chemical NLRP3 inflammasome inhibitors presents challenges that still need to be addressed in future clinical trials [125]. Regarding the possible link between diet, inflammasomes, and RA, it has been shown that fasting or a ketogenic diet can provide anti-inflammatory effects by increasing β-hydroxybutyrate levels, presumably via the inhibition of the NLRP3 inflammasome [126].

Inflammasomes, primarily the NLRP3 and AIM2 inflammasomes, are deeply involved in carcinogenesis [127]. However, unlike their pathogenic role in RA, their role in cancer development is controversial, as they exhibit both protective and enhancing effects [127]. The development of HPV-associated CC provides a good illustration of this point. A study of genetic polymorphisms in inflammasome-related genes (as possible risk factors for HPV infection susceptibility and CC progression) has revealed a statistically significant association between the NLRP3 variant rs10754558 and high-risk HPV resistance [128]. These data suggest that inflammasome activity (e.g., as determined by genetics) can impact HPV–host interactions by affecting virus susceptibility, virus persistence, and CC progression. However, it is not yet understood whether the inflammasome directly influences anti-viral or anti-tumoral activities. HPV16 activation of the AIM2 inflammasome in keratinocytes is accompanied by IL-1β, IL-18, and IFN-β secretion [129]. These data suggest that the source of IL-1β detected in the blood plasma of CC patients [114] may originate in immune cells rather than tumor cells. This implies that inflammation in keratinocytes may occur during the early stages of HPV infection, whereas immune system inflammation occurs in later stages. In cervical biopsies of HPV-infected women, lower IL-1β and IL-18 expression is associated with more than a two-fold increased risk of progression from CIN to CC [130]. This observation suggests that these cytokines play a protective (tumor-suppressive) role in the early stages of CC development. Cytosolic receptors, such as NLRP1, NLRP3, and AIM2, are activated during HPV infection of keratinocytes, presumably responsible for cellular defense against viral persistence [129]. Conversely, HPV-infected CC cells exhibit reduced AIM2 inflammasome expression, thereby suppressing pyroptosis and promoting cancer cell proliferation [131]. HPV possesses capacity to evade cellular defense mechanisms. For example, the HPV E7 protein can inhibit cell pyroptosis induced by double-stranded DNA (dsDNA) transfection [132]. The researchers show how the recruitment of E3 ubiquitin ligase TRIM21 by E7 causes ubiquitination and degradation of IFI16 inflammasomes. This process inhibits cell pyroptosis and allows viral evasion from immune surveillance. Additionally, HPV16 E6 binds to IL-18 in keratinocytes, promoting its degradation through ubiquitination, thereby interfering with the local IL-18-dependent downstream inflammatory cascade [133]. These data indicate that pyroptosis functions as a tumor suppressor in CC development, while HPV inhibits pyroptosis by repressing the expression of IL-1β, IL-18, and inflammasomes, thus promoting CC development. On the other hand, pyroptosis can create a suitable TME for tumor growth mainly by enhancing Granzyme B expression [10]. Therefore, the activation of inflammasomes and pyroptosis in the TME of CC might act as a double-edged sword in CC progression, as it has been proposed for other cancers [127]. In an early stage, inflammasome activation and pyroptosis can bring death to HPV-infected/transformed cells, attenuating cancer progression. However, in a later stage, when inflammasome activation and pyroptosis occur in the immune cells of the TME, this compromises the anti-tumor activity of the immune system, promoting cancer progression and metastasis. The diverse role of the NLRP3 inflammasome in the pathogenesis of CC makes the use of inflammasome inhibitors in CC questionable [127].

Nutrition has been repeatedly linked to HPV infection persistence, cervical neoplasia, and CC in case-control studies. Higher intake or serum levels of numerous nutrients, including vitamins A, C, E, B12, α-carotene, β-carotene, lutein, folate, zinc, and dietary fiber, have been linked to lower odds of CC in women [134,135,136,137]. Several mechanisms have been proposed to explain how these dietary factors could influence the course of HPV infection. These include antioxidant activity [138], increased production of retinoid acid, which is crucial in modulating epithelial cell growth and differentiation [139], and enhanced mucosal immune response to infection [140]. In a recent prospective observational cohort study, total and whole fruit and seafood/plant protein intake has been associated with HPV resolution using a logistic regression model [136]. However, a recent meta-analysis reveals that there is no significant association between the consumption of vegetables and fruits and the incidence of CC among studies controlled for HPV infection [141]. Although no available data exist on the relationship between nutrition, inflammasome activation, and CC, the beneficial effects of calorie restriction and low-fat diet on inflammasome-mediated processes in colorectal cancer [142] may suggest a similar relationship in CC.

## 8. Nutrition and the Role of the cGAS–STING Signaling Pathway in RA and CC Pathogenesis

The cGAS–STING pathway represents a novel therapeutic target for preventing and treating various diseases, including cancer and infectious and autoimmune diseases. The biochemical mechanisms of the cGAS–STING signaling pathway have been thoroughly investigated (e.g., [143]). In short, the binding of dsDNA results in conformational change and activation of cGAS. In turn, activated cGAS catalyzes the conversion of adenosine triphosphate (ATP) and guanosine triphosphate (GTP) to 2‘3‘-cGAMP, a cyclic dinucleotide (CDN). CDNs stimulate the adapter protein STING on the endoplasmic membrane, triggering phosphorylation of interferon regulatory factor 3 (IRF3) and activation of NF-κB. Effectively, this culminates in anti-tumor and antiviral type I interferon (IFN-I) responses. It is noteworthy that the hyperactivation of the cGAS–STING pathway and the resulting IFN-I responses are implicated in numerous inflammatory and autoimmune diseases. This makes cGAS–STING an attractive target for immunomodulation in the prevention medicine and therapy of various diseases.

The components of the cGAS–STING pathway can interact with TLR4, NF-κB, JNK, autophagy, AMPK, mTOR, and insulin signaling pathways, which collectively regulate inflammation and metabolism [144]. The activation of this pathway can also lead to fat accumulation in the adipose tissue, liver, and vascular endothelium, which can worsen conditions, such as obesity, nonalcoholic fatty liver disease (NAFLD), insulin resistance, and atherosclerosis [145,146]. Moreover, high-fat diet has been shown to increase cytosolic mitochondrial DNA (mtDNA) levels and activate the cGAS–STING signaling pathway in adipocytes, in concordance with heightened inflammation [147], thus linking nutrition to the regulation of this pathway. 

One of the hallmarks of RA pathogenesis is significant DNA damage in various immune cells [148,149,150] and fibroblast-like synoviocytes (FLSs) [151], accompanied by the accumulation of cytosolic dsDNA [152]. Cytosolic dsDNA activates cGAS–STING signaling that leads to the production of IFN-Is (IFNα and IFNβ) and inflammatory cytokines, thus linking DNA damage with inflammation in RA [153]. The role of IFN-Is in RA pathogenesis is less clear compared to the well-established detrimental role of pro-inflammatory cytokines (e.g., [154]). For example, intraperitoneal IFNβ administration in a mouse RA model delayed arthritis progression, involving the suppression of the NF-κB pathway [155]. Nevertheless, evidence also points to a pro-inflammatory role for IFN-Is in RA. This is based on their ability to stimulate monocytes and DCs and activate CD4+ and CD8+ T cells and antibody-producing B cells [156,157]. In line, elevated IFN-I signatures, identified by relatively high expression of IFN-I response genes (IRGs), have been shown to predict the development of RA in individuals at risk [158,159]. The induction of these IRGs is initiated by the dimerization and activation of the IFN-I receptors IFNAR1 and IFNAR2, which subsequently activate the JAK-STAT signaling pathway, also implicated in the pathogenesis of RA [160]. Before starting TNFi treatment, high serum IFN-I levels are predictive of therapy failure in RA patients [161]. Recent research indicates that the documented ability of TNFα to induce IFN-Is in RA [162] involves the activation of the cGAS–STING pathway in monocytes [12]. This study shows that TNFα inhibits mitophagy, resulting in mitochondrial dysfunction and an increase in cytosolic mtDNA, which then bind to cGAS and activate the cGAS–STING pathway. Another study shows that cytosolic dsDNA-induced cGAS/STING activation promotes FLS migration and invasion in conjunction with elevated mitochondrial ROS levels [106]. In support, significant cell-free dsDNA accumulation and STING activation have been detected in the synovial tissues of RA patients and mice of an RA model [163]. 

These observations indicate a negative role for the cGAS–STING signaling pathway in the pathogenesis of RA, suggesting that targeting this pathway could contribute to a successful treatment of RA. Indeed, the knockdown of cGAS or STING decreases cytosolic dsDNA-induced migration and invasion of FLS derived from RA patients [106]. In addition, in an inflammatory arthritis mouse model, cGAS deficiency blocks IFN-I responses and reduces inflammatory cell infiltration and joint swelling [12]. Small molecule inhibitors of the cGAS–STING pathway suppress FLS invasion into cartilage and inhibit RANKL-induced osteoclastogenesis, thus reducing the severity of RA [164]. In support, STING inhibition using a specific antagonist reduces the secretion of dsDNA-induced pro-inflammatory cytokines in macrophages. It effectively alleviates joint damage in both the dsDNA-induced and collagen-induced murine arthritis models [163]. Together, these promising results encourage the utility of cGAS and STING inhibitors in RA therapy, although clinical data are still lacking.

Although activation of the cGAS–STING signaling pathway plays a negative role in RA pathogenesis, its role in cancer remains clear. A number of in vivo studies imply that the cGAS–STING pathway may have protective effects against cancer. STING-deficient mice exhibit a higher susceptibility to tumor formation, reduced anti-tumor T cell immunity, and impaired responses to immunotherapy [165]. In contrast, elevated STING expression is positively associated with IFN-I levels and tumor-infiltrating CD8+ cytotoxic lymphocyte numbers [166]. However, STING activation can also enhance tumor metastasis by inducing the expression of epithelial–mesenchymal transition genes [167]. 

What is the function of the cGAS–STING pathway in HPV-related CC pathogenesis? In human CC cells, the HPV18 oncogene E7 disrupts cGAS–STING signaling, and E7 silencing restores it by selectively inhibiting STING-induced NF-κB activation and the nuclear translocation of p65. In addition, E7 inhibition of STING-induced NF-κB activation reduces HPV pathogenicity [13]. Furthermore, activation of the STING–TBK1 triggers the degradation of E7 oncoproteins through the ubiquitin–proteasome system, leading to the suppression of CC growth [168]. Noticeably, increased STING protein expression in CC tissue samples has been proposed as an independent prognostic factor for improved survival in both the surgery and radio(chemo)therapy CC groups [169]. These findings suggest that activation of the cGAS–STING signaling pathway could be a promising approach to treating CC, similar to pre-clinical studies of other solid cancers [170]. In accordance with this proposal, several STING agonists, including ADU-S100, E7766, and GSK3745417, have been approved for cancer therapy [171]. However, the efficacy of cGAS–STING agonists is so far limited due to challenges, such as poor drug stability, cellular toxicity, immune-related adverse events, and low cellular uptake [172].

## 9. Failed Resolution of Chronic Inflammation Is a Common Mechanism in RA and CC Pathogenesis

The present review outlines the emerging pathophysiological role of unresolved chronic inflammation in the pathogenesis of both RA and CC, emphasizing the importance of further research to unlock the potential therapeutic applications for these diseases and to improve their outcomes. The complex challenge of developing therapeutic applications is evident by the fact that these diseases are interconnected. Indeed, infection with HPV can aggravate the manifestations of RA, creating a vicious cycle of chronic inflammation. As a result, chronic inflammation can contribute to the enhanced progression of CIN to CC. Therefore, as suggested above, it raises the risk of developing CC in RA patients. According to our hypothesis, successful resolution of chronic inflammation could break this detrimental circle, leading to the alleviation of the symptoms in both diseases and reduced CC risk.

**Figure 3:**
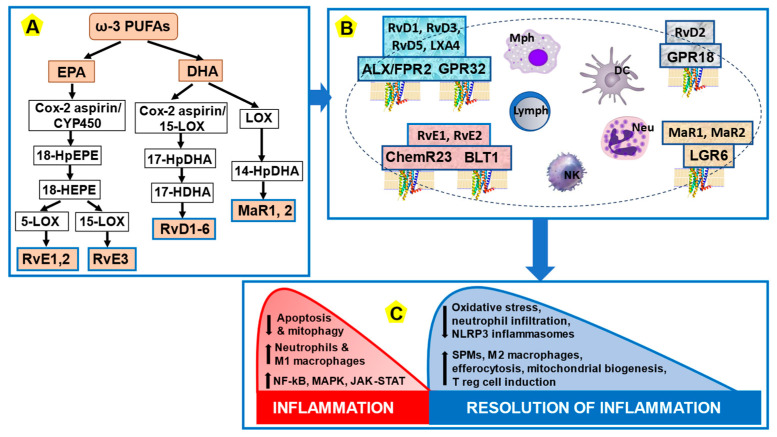
Simplified presentation of SPM biosynthesis, SPM receptors, and the process of inflammation resolution. (**A**) During the resolution phase of acute inflammation, SPMs (e.g., resolvins RvE1, 2, 3; RvD1-6) and maresins (MaR1,2) are biosynthesized from essential ω-3 PUFAs, including EPA and DHA. The main enzymes involved in the production of resolvins and maresins are CYP450 and LOX. There are several intermediates, such as 17/18-HpDHAs in the synthesis of resolvins and 14-HDHA in the synthesis of maresins by 12/15-LOXs. (**B**) SPMs trigger their pro-resolving signals via designated GPCRs expressed on various cells, mainly immune cells. (**C**) Physiologically, acute inflammation terminates by SPM-mediated resolution, which involves restriction of neutrophil tissue infiltration, counter-regulation of pro-inflammatory chemokines and cytokines, reduction of ROS and NLRP3 inflammasome generation, induction of apoptosis in active neutrophils and their subsequent efferocytosis by macrophages, accumulation of anti-inflammatory M2 macrophages, and other related processes. Ultimately, the resolution process leads to tissue healing and restoration of tissue homeostasis. Down arrow means “decrease”, up arrow means “increase”. Further explanations are given in the text. *Abbreviations*: GPCR, G protein-coupled receptor; EPA, eicosapentaenoic acid; HEPE, hydroxyeicosapentaenoic acid; HpDHA, hydroperoxydocosahexaenoic acid; HpEPE, hydroperoxyeicosapentaenoic acid; MAPK, mitogen-activated protein kinase; JAK-STAT, Janus kinase (JAK)-signal transducer and activator of transcription (STAT); LOX, lipoxygenase; Lymph, lymphocyte; Mph, macrophage; Neu, neutrophil; NK, natural killer cell; NF-κB, nuclear factor kappa-light-chain-enhancer of activated B cells; PUFA, polyunsaturated fatty acids; ROS, reactive oxygen species; SPMs, specialized pro-resolving molecules.

Inflammation resolution is a vital physiological process that protects tissues from prolonged or excessive inflammation that might cause tissue damage. In short, inflammation resolution is a process primarily driven by SPMs, such as lipoxins, resolvins, protectins, and maresins. These SPMs are derived from dietary PUFAs through the sequential enzymatic activities of lipoxygenases and hydrolases. For example, lipoxins (LXs), such as LXA4, are derived from the omega-6 PUFA arachidonic acid (AA). The omega-3 PUFA docosahexaenoic acid (DHA) serves as the substrate for D-series resolvins (RvDs), maresins (MaRs), and protectins, while eicosapentaenoic acid (EPA) serves as a substrate for E-series resolvins (RvEs) [173]. A key aspect of physiological SPM activity is the post-inflammation restoration of tissue homeostasis, which is elicited by tissue reparative/regenerative programs that act on both the innate and the adaptive immune systems [174]. SPMs exert their biological actions on cells via cognate G protein-coupled receptors (GPCRs), namely ALX/FPR2, GPR32, ChemR23, BLT1, GPR18, GRP37, and LGR6. These GPCRs are expressed by various immune cells, synovial fibroblasts, osteoclasts, and adipocytes. By binding to their receptors, SPMs trigger signaling pathways that promote the regeneration of inflammation-damaged sites, altogether establishing a beneficial equilibrium between pro-inflammatory and anti-inflammatory reactions. The major inflammation resolution mechanisms are schematically summarized in Figure 3.

It is apparent that the natural mechanisms of inflammation resolution in RA are suppressed [175,176]. These mechanisms have been under the scrutinous gaze of researchers (e.g., [174]). Several studies have shown that the development of RA is closely linked to a decrease in circulating SPM levels, often in correlation with disease severity [177,178]. In patients with early RA, serum lipid profiles rich in omega-3 PUFAs are independently associated with reduced disease activity [179]. It is widely accepted that chronic inflammation is a major component of ~25% of all human cancers [180]. Chronic inflammation can cause DNA damage and genomic instability, increasing mutational burden, and it can also promote the survival and proliferation of cancer progenitor cells [6,180,181]. Several human cancers have been shown to be associated with lower SPM levels [182], suggesting their involvement in the protection against cancer. Similarly to RA, the successful resolution of chronic inflammation may slow down CC progression and alleviate related symptoms. It has also been shown that dietary PUFAs, including DHA, can detect and attenuate NLRP3 inflammasome activation in human macrophages primed with LPS [183], and attenuate atherosclerosis development in a mouse model via inhibition of NLRP3 inflammasome activation [184]. These results suggest that the beneficial effects of dietary PUFAs in RA and CC patients may be mediated through suppression of inflammasome activation.

## 10. Application of Inflammation-Resolving Agents for Attenuating RA and CC Manifestations

Current RA therapy may include non-steroidal anti-inflammatory drugs (NSAIDs), corticosteroids, and conventional, biologic, and targeted synthetic DMARDs [185]. However, despite significant advances, RA therapy faces serious issues, including a lack of or partial response in a substantial proportion of patients, impaired tissue recovery/repair, poor pharmacokinetics, and adverse events due to non-selective immune suppression and off-target effects [186]. In this regard, it is noteworthy that SPMs facilitate the termination of the inflammatory response and initiate tissue repair and healing without inducing immunosuppressive effects [187]. Thus, SPMs have the potential to serve as a complementary approach to RA treatment. In support, dietary supplementation of omega-3 PUFAs (for at least 3 months at a dose of >2.7 g/day) increased EPA and DHA blood levels, but reduced omega-6/omega-3 ratio in association with decreased triglycerides and reduced tender joint count in RA patients [188]. Moreover, administration of various SPMs, mainly resolvins, in pre-clinical arthritis animal models attenuate disease manifestations [189]. Additionally, RvD1 [190] and MaR1 [191] significantly alleviate the pain associated with arthritis.

Current CC treatment consists of hysterectomy, chemo/radiotherapy, or their combination. In the case of standard therapy resistance, targeted therapy, mainly immunotherapy, is given to patients. Immunotherapies include therapeutic HPV vaccines, immune checkpoint inhibitors, engineered T cells, and antibody–drug conjugates [192,193,194,195,196]. The initial outcomes of ongoing clinical trials of immunotherapy in patients with advanced, persistent, or recurrent CC look promising [193,196]. However, this approach is still facing several challenges, such as high costs due to personalized treatment and selecting the suitable targeted antigen, long therapy duration, and serious side effects resulting from immunosuppression (e.g., risk of infections and cytokine storm syndrome) [197]. 

The consumption of fish and marine omega-3 PUFAs has shown an evident protective effect on the survival of cancer patients, according to a recent meta-analysis of 21 cohort studies [198]. In pre-clinical cancer models, SPMs, mainly resolvins and lipoxins, successfully resolve chronic inflammation, supporting the idea that they may offer a new approach to cancer treatment [182,187,189,198,199,200]. Of interest, RvD2, RvD3, and RvD4 induce anti-tumor T cell response and inhibit metastasis in various cancer models [201]. Another study shows that RvD1, RvD2, or RvE1 inhibit chemotherapy-induced cancer progression by improving debris clearance through macrophage phagocytosis in multiple tumors [199]. In addition, these resolvins reduce the release of pro-inflammatory TNFα, IL-6, IL-8, CCL4, and CCL5 by human macrophages stimulated with cell debris. These data indicate that SPMs may have a dual function in cancer-associated inflammation: inhibiting the production of pro-inflammatory cytokines and simultaneously activating macrophage phagocytosis of pro-tumorigenic cellular debris, thereby resolving chronic inflammation that drives tumor growth. In addition, activation of endogenously produced lipoxins and resolvins may offer a new approach to cancer therapy through cell-autonomous and non-cell-autonomous mechanisms in the TEM. The involved mechanisms include NF-κB pathway inhibition [202], regulation of the miR-138-p5/Forkhead box C1 protein (FOXC1) pathway [203], MAPK pathway inhibition [204], modulation of macrophage polarization [205], cancer-associated fibroblasts-derived cartilage oligomeric matrix protein (COMP) inhibition [206], and reduced TNFα-induced c-Myc expression [207]. With regard to HPV-associated carcinogenesis, RvD1 has been shown to reduce the proliferation of HPV-positive human and mouse CC cells. This occurs by directly stimulating the anti-tumor activity of neutrophils and enhancing the recruitment of anti-cancer monocytes [208].

Yet, it is worth mentioning that SPMs are rapidly degradable molecules, so more stable analogues and receptor agonists have been synthesized and tested in various animal models of inflammation [173,209]. For example, the stable epimer 17R-RvD1 and the ALX/FPR2 mimetic BML-111 significantly decrease arthritis severity and shorten the remission interval in murine inflammatory arthritis [210,211]. Of note, BML-111 has been shown to inhibit the development of hepatocarcinoma [212] and melanoma [213]. The recently developed LXA4 mimetic NAP1051 demonstrates anti-tumor activity in a colorectal cancer xenograft model [214]. Taken together, these results suggest that using SPM mimetics and receptor agonists in RA and cancers associated with chronic inflammation could improve current anti-RA and anti-cancer therapies. In the case of HPV-infected RA patients, the application of SPM mimetics and receptor agonists has the potential to attenuate or resolve chronic inflammation, leading to a reduced risk of CC development.

## 11. Concluding Remarks

In this review, we presented evidence supporting the hypothesis that the failure to resolve chronic inflammation underlies the increased risk of developing CC in patients with RA. The challenge lies in determining whether this concept can be useful for developing new therapeutic approaches to reduce the risk of CC in general. Recent years have seen a revolutionization in the treatment of both pathologies with the introduction of immunotherapy, including immune checkpoint inhibitors and adoptive cell therapies. However, these therapies have several serious drawbacks, which often limit their use, such as acquired resistance and immune-related adverse effects. Furthermore, despite the profound involvement of activated inflammasomes and the cGAS–STING signaling pathway in the pathogenesis of both RA and CC, their applicable targeting remains unsatisfactory. 

Many clinical observations report that different nutritional approaches, such as certain diets (e.g., Mediterranean diet), calorie restriction, and the supplementation of PUFAs, polyphenols, and vitamins, have beneficial effects on the manifestations of conditions like RA, HPV infection, and various cancers, including CC. Interestingly, it has been suggested that an “ideal meal” for the care and management of RA should include raw or moderately cooked vegetables, with the addition of spices like turmeric and ginger, seasonal fruits, and probiotics, all of which are good sources of natural antioxidants [215]. Similar recommendations are offered as an adjunctive cancer treatment [216]. Importantly, despite the lack of compelling confirmation in comprehensive meta-analyses, these nutritional approaches have been shown to alleviate the symptoms of chronic inflammation.

The present review suggests the use of stable SPM mimetics and receptor agonists, which demonstrate beneficial effects in numerous pre-clinical models of arthritis and tumors. This has the potential to address the shortcomings of traditional therapies and current immunotherapies for RA and cancer. The critical distinction between pro-resolving mediators and anti-inflammatory mediators lies in the fact that while most anti-inflammatory agents can cause immunosuppression, the resolution of inflammation occurs through active endogenous reprogramming of the immune response, allowing the termination of inflammation without causing immunosuppressive effects.

**Figure 4:**
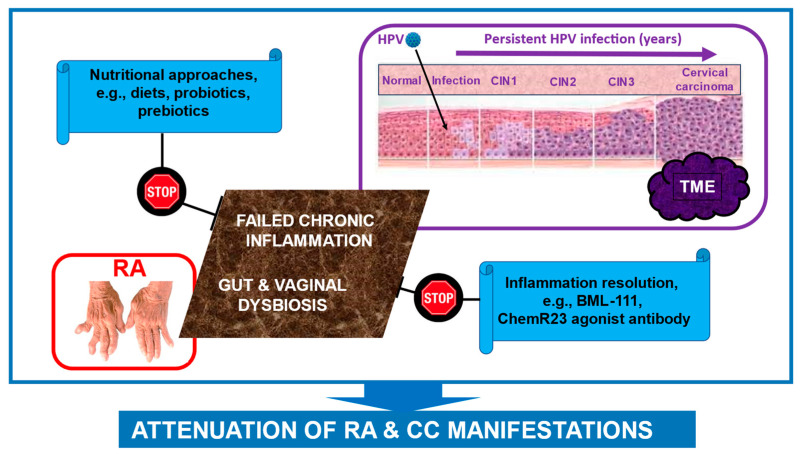
Proposed therapeutic strategy to attenuate the manifestations of RA and CC and reduce CC risk in patients with RA by reversing chronic inflammation and correcting dysbiosis. We assume that failure to resolve chronic inflammation, as well as gut and vaginal dysbiosis, are key factors in the pathogenesis of RA and CC, aggravated manifestations, and increased risk of CC development in RA patients. These processes appear to mutually interact, creating a vicious cycle that maintains and worsens both RA and CC. Accordingly, the use of stable SPM receptor mimetics and agonists, such as BML-111, the anti-ChemR23 agonist antibody (resolvin E1 receptor), and dysbiosis correction have promising therapeutic potential for these conditions. Those treatments are intended to complement traditional treatments for RA and CC. Further explanations are given in the text. *Abbreviations*: HPV, human papillomavirus; SPMs, specialized pro-resolving molecules; RA, rheumatoid arthritis.

A new page in this clinical area was opened by studies of an agonist antibody against ChemR23. Via induction of SPM receptor signaling, its mode of action involves accelerating acute inflammation resolution, promoting macrophage efferocytosis, and reducing neutrophil apoptosis at the site of inflammation [217]. Remarkably, this antibody decreases lung metastasis and increases animal survival in a pre-clinical model of aggressive triple-negative breast tumors, after the resection of the primary tumors. The results are correlated with the modulation of tumor-associated macrophages in the metastatic niche [218]. Based on these findings, we propose a novel, combinatory treatment strategy to alleviate RA and CC manifestations. For simplicity’s sake, this strategy is summarized in Figure 4 and includes implementing healthy nutritional approaches alongside SPM mimetics and receptor agonists as a potential therapeutic modality to resolve chronic inflammation. This treatment strategy can be used in conjunction with traditional or modern treatments for RA and CC. However, the implementation of this proposed combinatory therapeutic strategy requires overcoming critical research challenges. What are the dominant factors that contribute to chronic inflammation in HPV-associated CC? Is the relationship between these two pathogenic processes causal, or do they rather represent independent pathological mechanisms? Could SPM mimetics and receptor agonists be clinically effective in RA patients with CC? Obviously, substantial information about the specificity, safety, tolerability, side effects, and effectiveness of this complementary approach is warranted. How can early signs of chronic inflammation be detected in RA patients suffering from CC? Is it possible to categorize these patients based on their microbiota composition and the degree of chronic inflammation? If so, identifying and characterizing these profiles could offer a valuable opportunity to categorize patients from a personalized medicine standpoint. This could provide further insight into the mechanisms of increased CC risk in RA patients. We believe that the increased CC risk in RA patients can be primarily attributed to the failure of chronic inflammation resolution and dysbiosis. Accordingly, using pro-resolving compounds and dysbiosis correction approaches have the potential to effectively reduce this risk. In order to harness the benefits of the proposed multi-level prevention/treatment strategy, the crucial next step is to conduct scientific search for highly specific targets (receptors, cells, and microbes). However, there is currently a vast variety of possible targets, making the development of such a complex therapeutic strategy a highly challenging task. Once we overcome this challenge, it will open the door to developing personalized treatments for HPV-infected RA patients to significantly reduce manifestations and the risk of developing CC.

## Figures and Tables

**Table 1:** Summary of the recent epidemiological studies of CC in RA patients.

Source *	N/n	Statistical Indicator
E. Foster et al., 2020 [39]	1246/25 (CIN 1)	AHR 1.23 (95% CI 1.05–1.42)
H. Wadström et al., 2016 [4]	44,613/212 (CIN 2+)	HR 1.39 (95: CI 1.16–1.66)
P. Dugué et al., 2015 [38]	56,142/140 (CC)	SIR 1.1 (95% CI 0.9–1.2)
M. Beydon et al., 2023 [1]	189,335/332 (CC)	SIR 1.80 (95% CI 1.62–2.01)
S. Kim et al., 2014 [3]	58,979/818 (CIN 2 + CC)	HR 1.49 (95% CI 1.11–2.01)
H. Lee et al., 2019 [5]	1586/9 (CC)	SIR 3.65 (95% CI 1.65–6.42)

The studies provided in the table show profound differences in the number of enlisted patients with RA (ranging from 1246 to 189,335 patients) and CC (9–332 cases) and were conducted in several countries (Australia, Sweden, Denmark, USA, France, and Korea). The provided studies in the table clearly demonstrate a consistent trend for RA–CC positive association in the past decade. * The studies in the table are listed as they appear in the text. Three studies use SIR as a statistical measure, two use HR, and one uses AHR. Abbreviations: N, number of patients with rheumatoid arthritis (RA); n, number of patients with studied phenotype; AHR, adjusted hazard ratio; HR, hazard ratio; SIR, standardized incidence ratio; CIN 1, cervical intraepithelial neoplasia 1, corresponding to low-grade squamous intraepithelial lesion cytology; CIN 2+, cervical intraepithelial neoplasia 2, 3, corresponding to high-grade squamous intraepithelial lesion; CC, cervical cancer.

## Data Availability

No data were used for the research described in the article.

## List of Abbreviations

**Acronym**	**Explanation**
ATP	Adenosine triphosphate
CC	Cervical cancer
CDN	Cyclic dinucleotide
cGAMP	Cyclic guanosine monophosphate–adenosine monophosphate
DCs	Dendritic cells
CIN	Cervical intraepithelial neoplasia
DMARDs	Disease-modifying antirheumatic drugs
dsDNA	Double-stranded DNA
FLSs	Fibroblast-like synoviocytes
GALT	Gut-associated lymphoid tissue
GPCRs	G protein-coupled receptors
GTP	Guanosine triphosphate
HPV	Human papillomavirus
HR	Hazard ratio
IFN-I	Interferon type I
IRGs	IFN-I response genes
JAK-STAT	Janus kinase/signal transducers and activators of transcription
LRR	Leucine-rich repeat
LxCxE	Leucine-X- cysteine-X-Glutamic acid X is any amino acid
MAPK	Mitogen-activated protein kinase
MaR	Maresins
MDSCs	Myeloid-derived suppressor cells
MMPs	Metalloproteinases
mtDNA	Mitochondrial DNA
NAFLD	Nonalcoholic fatty liver disease
NF-kB	Nuclear factor kappa B
NSAIDs	Non-steroidal anti-inflammatory drugs
PUFAs	Polyunsaturated fatty acids
RA	Rheumatoid arthritis
RvE	E-series resolvins
RvD	D-series resolvins
SCFAs	Short-chain fatty acids
SFs	Synovial fibroblasts
SIR	Standardized incidence ratio
SNPs	Single nucleotide polymorphisms
SPMs	Specialized pro-resolving mediators
TAK1	TGF-β-activated kinase 1
TNFi	Tumor necrosis factor inhibitor
TLR	Toll-like receptor
TME	Tumor microenvironment
URR	Upstream regulatory HPV region
